# Balancing chemical safety and animal welfare considerations in the application of new approach methodologies for chemical safety assessment

**DOI:** 10.1016/j.namjnl.2025.100013

**Published:** 2025-03-01

**Authors:** Aleksandra Čavoški, Laura Holden, Leonie Mueller, Robert Lee

**Affiliations:** a Birmingham Law School, University of Birmingham, Edgbaston, Birmingham, B15 2TT, UK; b Altertox, Alphonse de Witte 11, 1050 Ixelles, Belgium

**Keywords:** NAMs, NGOs, Consensus

## Abstract

Despite some considerable advancement in developing different forms of new approach methodologies (NAMs) to assess the risk of chemicals, there is still limited progress in their application for regulatory purposes in the European Union (EU). Distinct divisions exist between non-governmental organisations (NGOs) with an animal welfare agenda, and those focusing on environmental protection. The conflicting views on NAMs held by these NGOs are a point of concern for policy makers in particular. This report gathers and reviews the perspectives of selected animal welfare and environmental NGO representatives who responded to our small-scale, anonymous online survey. The responses were analysed to identify areas where there is some space to accommodate the views of both groups. There exists strong consensus on the question of whether EU chemical legislation on risk assessment is sufficiently well adapted to incorporate new approach methodologies. Both NGO groups do acknowledge the merit of scientific advances to aid decision making, albeit with the cautious need to ensure that the information to truly determine the safe use of chemicals is available. Broad consensus was identified: i) for the need of a more precise definition of NAMs by making reference to using NAMs in specific contexts; ii) acknowledging that existing processes are not adequate to validate new approach methodologies; and iii) that there remain clear barriers to the regulatory deployment of NAMs. Finally, moderate consensus was identified for the question of whether animals are an effective and appropriate surrogate to assess the impact chemicals have on environmental health, with both groups acknowledging, to an extent, that animal tests in this context can be useful indicators and NAMs could assist in identifying further effects in complex systems. Understanding these areas of common ground will benefit future policy decision-making related to the acceptance of NAMs in chemical safety assessments.

## Highlights

• Survey reveals areas of consensus between animal welfare and environmental protection NGOs on utilization of new approach methodologies (NAMs) to assess the risk of chemicals.
• Broad consensus was identified for the need of a more precise definition of NAMs by making reference to using NAMs in specific contexts, acknowledging that existing processes are not adequate to validate new approach methodologies and that there remain clear barriers to the regulatory deployment of NAMs.
• Understanding these areas of common ground will benefit future policy decision-making related to the acceptance of NAMs in chemical safety assessments.

## 1 Introduction

Although significant progress has been made in developing new approach methodologies (NAMs) to assess the safety of chemical, their use for regulatory purposes in the European Union (EU) remains limited. Empirical research within the PrecisionTox programme explored the barriers to this regulatory uptake of NAMs from the viewpoints of industry, regulators, and policy makers (Čavoški et al., 2023). In the course of that research, it became clear that conflicting views on NAMs held by non-governmental organisations (NGOs) were a point of concern for policy makers in particular. NGOs have the potential to voice disquiet in an organised campaign citing science and by actively participating in the policy making process. Respondents in the barriers research reported engaging with opposing camps of NGOs, some supporting and advocating a wider ban on animal testing while others urging caution in the face of strong concerns about levels of safety if NAMs were to become more widely used. This has a potentially inhibiting effect where the policy maker seeks to reach a consensus on NAMs, and this may impact at the very least upon the timetable for animal free chemical safety assessment, to which the European Commission is committed (EU Commission DG GROW, 2023).

This study gathered and reviewed the perspectives of relevant civil society organisations and the underpinning research sought to identify points of agreement between them, as such areas of consensus will help inform future policy decision-making related to the acceptance of NAMs in chemical safety assessments. From the position statements and other published views by NGOs, we were able to identify some specific NGOs with differing agendas in the NAM debate. For this study, we classified these NGOs into two broad groups: those with an animal welfare agenda, and those focusing on environmental protection either specifically relating to chemical pollution or more widely.

## 2 Material and methods

### 2.1 Analysis of NGOs public statements

Using a set of defined search terms (Table 1), the publicly available official communication of selected NGOs (Table 1), with known expertise and public positions on animal testing for chemical safety assessments was collected.

**Table 1:** Search terms and NGOs included in the online search of public articles on animal testing for chemical safety assessment.

Search Term	NGOs/ coalitions
Animal testing	European Animal Research Association
Animal experimentation	People for the Ethical Treatment of Animals
ECHA	The Humane Society
REACH	Eurogroup for Animas
REACH animal testing	Cruelty Free International
Chemical	The European Coalition to End Animal Experiments
Chemical safety	Doctors Against Animal Experiments
Toxicity testing	Faunanalytics
Laboratory animals	European Environmental Bureau
Rabbits	CHEM Trust
Rats	GMWatch
Animals	Health and Environment Alliance
	Pesticide Action Network Europe

### 2.2 Discussion group and survey

During an initial meeting in Brussels, the general perceptions of NAMs were discussed with representatives of the two NGO groups to help map out main issues raised by both groups in regards to the use of NAMs for chemical safety assessment. Participants were assured that the discussion group was to be conducted under the ‘Chatham House’ rule. Under this rule, participants in a meeting are free to use information received in that meeting but neither the identity nor the affiliation of any participant in the meeting, including the speaker providing the information, may be disclosed. Members of both NGO groups, identified to be involved in the NAM debate, were invited to complete a small-scale, anonymous, online survey. Through the survey, participants were able to express the reasoning behind the positions adopted in relation to specific aspects of the wider NAM debate, including: the definition of NAMs; barriers to the uptake of NAMs; the utility of NAMs; the human and environmental relevance of NAMs; and their validation (full list of questions in Supplement Table S1). The outcome is a more nuanced understanding of what, at first sight, appear to be opposing views (Section 2) such that Section 3 is able to draw out areas in which a degree of consensus becomes apparent following analysis. We conclude by summarising the areas of common ground and seek to show insight into these, which might benefit policy decision making.

## 3 Results & discussion

The following sections presents and discusses the research data of both, the analysis of public statement about NAMs in chemical safety assessment and the survey results, in an attempt to classify the views of the NGO groups in relation to NAMs. While there is a limitation to this empirical analysis in that our sample sizes are small, the weight to the conclusions drawn on the points of consensus are supported by a much wider ranging desk-based documentary evaluation of position statements on this issue, which are already in the public domain.

### 3.1 Analysis of NGOs public statements

In total, 60 articles were found and analysed regarding the organisation's position on NAMs.

The position statements, press releases, reports, and consultation responses published by the NGO groups do indicate differences in their views regarding the adoption of NAMs in chemical safety assessment. Animal welfare NGOs tend to point to the shortcomings of some animal tests, such as poor reproducibility and limitations for human relevance, and contrast this to the data derived from NAMs (People for the Ethical Treatment of Animals, n.d.). Others also note the time and cost inefficiencies of animal testing, especially when placed against the large number of chemicals requiring evaluation with which NAMs might assist (Humane Society International, n.d.). Animal welfare NGOs also regularly inform their stakeholders about international developments in the regulatory implementation of NAMs, such as South Korea's prioritisation of alternative methods over animal tests (Humane Society International, 2018), and the banning of animal testing on cosmetics in Canada. Additionally, they promote the availability of alternative methods to replace animal tests (Cruelty Free International, n.d.); celebrate successes in promoting legislative change (The European Coalition to End Animal Experiments, n.d.); and monitor and report on animal use statistics (Doctors Against Animal Experiments, 2023).

Environmental NGOs, on the other hand, express ‘deep concern, at the implications for public health and biomedical research, of calls by animal groups for an EU roadmap to phase out all animal research’ (European Animal Research Association, 2023a). Some are concerned that a move away from animal testing would diminish the life sciences sector in Europe (European Animal Research Association, 2023b), while others fear a dilution of safety as ‘available in vitro/silico methods are not yet sufficiently predictive,’ particularly for complex endpoints such as the endocrine system (European Society of Endocrinology, n.d.; GMWatch, 2021). There is a spectrum of views in this group: some feel that the promotion of NAMs over animal tests is a tool to allow the chemical industry to evade scrutiny (GMWatch, 2017) and that their use should be prohibited (Pesticide Action Network Europe, 2016), whereas others recognise the usefulness of some NAMs in screening so that the number of substances to be tested in vivo are reduced, while providing more information on modes of action (European Society of Endocrinology, n.d.; GMWatch, 2021).

The views about human relevance of animal testing when assessing the impact of chemicals were further explored in the survey and following documentary analysis. Whether animals are a good and appropriate surrogate to assess the impact chemicals have on human health were unsurprisingly split along the NGO group lines: animal welfare NGOs unanimously disagreed (citing, for example, differences in responses across species), whereas environmental NGOs were firmly in agreement that animals are an effective surrogate for this purpose. The latter group acknowledged there are limitations with animal testing, but noted animals provide whole-organism information, which NAMs do not. In some cases, there was acceptance of the utility of NAMs in screening, but in a somewhat guarded fashion due to concerns of industry bias.

Beyond the above points of dispute, there are gradations of views held by NGOs, which at times intersect such that levels of agreement can be discerned. The next section describes these areas based on the results of the survey, identifying a point of strong consensus, three areas of broad agreement from the survey, and one of moderate extent.

### 3.2 Discussion group and survey

#### 3.2.1 Strong consensus

##### 3.2.1.1 EU chemical legislation on risk assessment is not sufficiently well adapted to incorporate new approach methodologies

Previous research of other stakeholder groups had identified that the legal framework of the EU is felt to be a barrier to the uptake of NAMs (Čavoški et al., 2023; Sauer et al., 2017; Zaunbrecher et al., 2017). The current survey with NGO representatives revealed unanimous agreement that the current EU chemical legislation on risk assessment is insufficiently well adapted to incorporate NAMs. Respondents noted that although legislation recognises and encourages the principle of last resort (European Parliament and Council, 2006), in practice this is reported to be poorly applied due to regulatory hesitancy in embracing new methods. Frameworks are considered to be rigid and hazard centric, with information requirements in the EU being specific and prescriptive. The value and therefore the need to be able to incorporate informative mechanistic, molecular, and biologically relevant data rather than data derived purely from endpoints observed in animals, was acknowledged. The merit of scientific advances to aid decision making appears to be appreciated across respondents, albeit with the cautious need to ensure that the information to truly determine the safe use of chemicals is available.

#### 3.2.2 Broad consensus

##### 3.2.2.1 A more precise definition of NAMs is needed

Reference to NAMs in legislation is by way of terminology of non-animal or alternative methods. Some lower-tier endpoints in legislation can be informed by non-animal methods and are stated in, for example, the REACH annexes. More widely, many definitions of the term ‘NAMs’ exist and indeed for the animal welfare NGOs the term refers to ‘non-animal methods.’ There was broad consensus in the survey responses that a more precise definition of NAMs is needed, for example, by making reference to using NAMs in specific contexts, such as specifying a particular in vitro or in silico method used for a specific hazard assessment. A main caveat in defining NAMs comes from the fact that this concept fluctuates over time and what is used today may become outdated as science continues to develop in future. Indeed, it was pointed out that some NAMs have already been used over a significant period of time and thus it may be more appropriate to refine the notion of a ‘new’ methodology. Additionally, identifying those methods that are truly ‘non-animal methods,’ and which models and methods have human relevance were also suggested as a better approach. A recent publication by the Animal-free Safety Assessment of Chemicals: Project Cluster for Implementation of Novel Strategies (ASPIS) provided an inclusive NAMs definition, intended to help guide this discussion (Colbourne et al., 2025).

##### 3.2.2.2 Existing processes are not adequate to validate new approach methodologies

Validation of test methods has been identified as a critical aspect to the regulatory acceptance of NAMs during previously conducted research with other stakeholder groups (Batista Leite et al., 2021; Čavoški et al., 2023; Mondou et al., 2020; OECD, 2024; Sauer et al., 2017; Zaunbrecher et al., 2017). Animal welfare NGOs point to some animal tests as never having undergone validation but do accept that validation supports approaches now that seek to replace, reduce, and refine animal testing (3Rs). Environmental NGOs are concerned that validation must accredit the level of safety provided by animal tests. Thus, the assurance of relevance and reliability of tests afforded through validation is clearly important to both groups. There was also broad consensus from the survey respondents that existing processes are not adequate to validate NAMs. It was noted that validation procedures should be fit-for-purpose and relevant to the scientific methods and the endpoint being assessed, rather than being evaluated against the data from animal tests, which may not be, for example, directly relevant to humans. Although it was noted that validation is time-consuming and expensive, it was suggested that NAMs with advanced readiness could have their validation prioritised, particularly those identifying molecular changes.

##### 3.2.2.3 There remain clear barriers to the regulatory deployment of NAMs

Earlier research with other stakeholder groups identified several sociotechnical barriers to the uptake of NAMs (Batista Leite et al., 2021; Čavoški et al., 2023; Mondou et al., 2021, 2020; Sauer et al., 2017; Vachon et al., 2017; Zaunbrecher et al., 2017). These include concerns of the scientific maturity of NAMs, such as the lack of comprehensive biological coverage of NAMs for complex endpoints and a need to understand and accept the use of mechanisms of adverse outcomes in place of visible apical harms. There are concerns of the time taken to achieve the consensus needed for validation and whether assessments for validation are fit-for-purpose, such as with reference to human relevance. Views of regulatory science and the legislative framework noted a lack of acceptance by regulators; of less comparative familiarity and confidence with NAM science and technology than with animal studies; and a lack of trust between actors in the regulatory process. The need for expertise and resources also adds pressure to regulatory capacity and this may limit the ability to gain experience with NAMs. It was also noted by survey respondents that the general public is relatively unaware of the use of animal testing beyond cosmetics, along with fears that chemical safety may be diluted. Finally, the regulatory objectives of a jurisdiction were also considered to impact the acceptance of NAMs, due to differences in whether the principles of hazard or exposure underpin the legal framework on industrial chemicals.

These thematic barriers identified from previous empirical research as impacting the uptake of NAMs were widely agreed on by the NGO survey respondents to be the main themes. Additional barriers that these NGO participants highlighted include the lack of funding and coordination of NAMs for regulatory relevance.

#### 3.2.3 Moderate consensus

##### 3.2.3.1 Animals are a good and appropriate surrogate to assess the impact chemicals have on environmental health

Ecotoxicology and environmental protection are areas in which one might expect alignment on the protection goals of chemical legislation among these NGO groups, as both are truly concerned by species loss. Current risk assessment based on tests with a limited group of selected model organisms does not fully reflect the complexity of species and their interactions in the environment. This leaves data gaps in the assessment of the environmental impact of chemicals that NAMs could aid in filling. Although the appropriateness of animals as surrogates for human health remains a highly disputed area, utilizing animals as a surrogate for environmental health was slightly less controversial among the NGO representatives and is therefore an area in which greater consensus might be built. For example, environmental NGOs were broadly supportive of the statement, although this was not without limitations, which were also raised by the animal welfare NGOs. The complexity of the environment with its inter-relationships means it is not possible to test the impact of chemicals on many different organisms and systems that are representative of the environment. It was conceded that animal tests are therefore useful as indicators for this type of risk assessment and some respondents outlined that there are good model organisms to assess the environmental impact of chemical pollutants (such as bees). While animal tests may act as indicators, it was suggested that in silico NAMs, the use of NAMs in screening, and field studies could all assist in identifying further effects in complex systems, rather than relying on studies of relative isolation.

## 4 Conclusion

There exist distinct divisions between the views of animal welfare and environmental NGO groups on balancing chemical safety and animal welfare considerations in the application of NAMs for chemical safety assessment, particularly on the utility of animal tests in assessing the impact that chemicals have on human health. Nevertheless, our research has identified areas in which there is some space to accommodate the views of both groups. Notwithstanding different aspirations for the use of NAMs, broadly both groups come together in agreement that the robust nature of the science should drive advances in chemical safety assessment. From the areas where common ground has been identified, the following list demonstrates areas in which there is a level of agreement between animal welfare and environmental NGOs. This understanding of general consensus may prove helpful to both policy makers and NGOs in broking agreement in the future. This study is also timely as it may further inform the development of the European Commission's activities on developing a roadmap to phase out animal testing in chemical safety assessments:

• EU chemical risk assessment legislation requires adapting to better incorporate the consideration of reliable and relevant data from NAMs.
• References to ‘NAMs’ need to be refined by the specific context in question, including the method of science or technology, and in relation to any animal data or human relevance.
• Validation is important but procedures must be fit-for-purpose, such as in determining human relevance and as suitable for science and technology that continues to develop.
• The barriers to be overcome for the uptake of NAMs relate to their scientific maturity; validation; the legislative framework; expertise and resources; social perceptions; regulatory objectives; funding; and the coordination of methods for regulatory relevance.
• The application of NAMs in exploring eco-toxicity and to ensuring the protection of environmental health is acknowledged and supported by both groups.

The findings of this research compliment well the wider set of recommendations for better deployment of NAMs incorporated in the PrecisionTox ‘Solutions Report’ (Čavoški et al., 2024). The animal welfare and environmental NGO communities are highly informed about chemicals legislation in the EU, care deeply about the issues at hand, and wish to participate in the policy forum. This research has sought to draw on their knowledge and experience in a positive way, to uncover common rather than contrasting goals. Our research across the stakeholder groups has highlighted the importance in creating an inclusive process to bring all stakeholders together in assessing the role that NAMs can play in the regulatory process.

## CRediT authorship contribution statement

**Aleksandra Čavoški:** Funding acquisition, Investigation, Methodology, Project administration, Supervision, Writing – original draft. **Laura Holden:** Conceptualization, Data curation, Investigation, Methodology, Writing – original draft. **Leonie Mueller:** Conceptualization, Validation, Writing – review & editing. **Robert Lee:** Conceptualization, Investigation, Writing – original draft, Writing – review & editing.

## Declaration of competing interest

The authors declare that they have no known competing financial interests or personal relationships that could have appeared to influence the work reported in this paper.

## Data Availability

The data that has been used is confidential.

## Appendix Supplementary materials

Image, application 1
