# Supplementary material for: Balancing chemical safety and animal welfare considerations in the application of new approach methodologies for chemical safety assessment
Source: NAM J. 2025 Mar 1;1:100013. doi: 10.1016/j.namjnl.2025.100013 (PMC13289033; doi:10.1016/j.namjnl.2025.100013)
Supplement: Supplementary file 1 [file mmc1.docx]

Supplement Table S1: Online survey questions sent to animal welfare and environmental protection NGO representatives.

| No | Question | Type |
| --- | --- | --- |
| 1 | Is the remit of your organisation primarily concerned with: | Single choice:   1. Animal welfare 2. Environmental protection |
| 2 | Is a more precise definition of NAMs needed? | Open text |
| 3 | Do you feel this is comprehensive list of the barriers to the uptake of NAMs: Scientific maturity of NAMs; Validation; Legislative Framework; Expertise and Resources; Social Perceptions; and Regulatory Objectives (jurisdiction differences such as a focus of hazard vs risk)? | Open text |
| 4 | In your view, are current EU chemical legislation on risk assessment sufficiently well adapted to incorporate new approach methodologies? | Open text |
| 5 | Statement: Animals are a good and appropriate surrogate to assess the impact chemicals have on human health. Agree or disagree? | Open text |
| 6 | Statement: Animals are a good and appropriate surrogate to assess the impact chemicals have on environmental health. Agree or disagree? | Open text |
| 7 | Are existing processes adequate to validate new approach methodologies? | Open text |
